# Benefits of Escin for Decompression Sickness in Bama Pigs by Endothelial-Targeting Protection

**DOI:** 10.3389/fphys.2019.00605

**Published:** 2019-05-21

**Authors:** Long Qing, Wentao Meng, Wei Zhang, Hong-jie Yi, Kun Zhang, Dinesh K. Ariyadewa, Wei-gang Xu

**Affiliations:** ^1^Department of Diving and Hyperbaric Medicine, Naval Medical University, Shanghai, China; ^2^Department of Medicine, Sri Lankan Navy, Colombo, Sri Lanka

**Keywords:** decompression sickness, horse chestnut seed, escin, swine, endothelial dysfunction

## Abstract

Endothelial dysfunction has been considered as pivotal in the pathogenesis of decompression sickness (DCS) and contributes substantively to subsequent inflammatory responses. Escin is well known for its endothelial protection and anti-inflammatory properties, and its protection against DCS has been proved in a rat model. This study aimed to further investigate the protection of escin against DCS in swine. Sixteen swine were subjected to a two-stage experiment with an interval of 7 days. In each stage, 7 days before a simulated air dive, the swine were treated with escin or saline. The first group received a successive administration of escin for 7 days prior to the first dive and saline for 7 days prior to the second; the second group was treated with saline and then escin. After decompression, signs of DCS and circulating bubbles were monitored, and blood was sampled for platelet count and determination of inflammatory and endothelial related indices. The death rate of DCS was markedly decreased in swine treated with escin compared with that in animals treated with saline, though not statistically significant due to the limited number of animals. Escin had no effect on bubble load but significantly ameliorated platelet reduction and endothelial dysfunction, as well as oxidative and inflammatory responses. The results further suggest the beneficial effects of escin on DCS by its endothelia-protective properties, and escin has the potential to be a candidate drug for DCS prevention and treatment.

## Introduction

In sudden and unexpected events during diving, aviation, and other barometric pressure exposures, an excessively rapid drop of ambient pressure can induce the formation of inert gas bubbles, which will entail a serious risk of decompression sickness (DCS) (Vann et al., 2011). Bubbles are at the core of the pathogenesis of DCS by mechanical injury and their secondary reaction with vascular endothelia leading to inflammation and vascular dysfunction (Brubakk and Møllerløkken, 2009). We have confirmed that endothelial dysfunction induced by decompression was well correlated with bubble formation in a rat model (Zhang et al., 2016). It has been proven in our previous studies that drugs with potential endothelial protection, such as simvastatin, were beneficial to DCS (Zhang et al., 2015). Escin is an endothelium-protective drug that is extracted from *Aesculus hippocastanum* seeds. Escin can reduce vascular permeability, increase venous reflux, alleviate venous congestion symptoms, increase vascular elasticity and tension, and reduce oxygen free radicals (Sirtori, 2001; Patlolla and Rao, 2015). Clinically, escin is widely used in treating chronic venous insufficiency, soft tissue swelling, cerebral edema, and so on (Domanski et al., 2016). In our previous study, escin was found effective at preventing DCS in rats, mostly *via* endothelial protection (Zhang et al., 2017a).

This study aimed to further prove the effects of escin on DCS in a swine model. Circulating bubbles, skin lesions, platelet count, and inflammation and endothelial injury markers were observed to investigate the effects of escin upon DCS incidence.

## Materials and Methods

### Animals

Sixteen neutered male Bama swine at the age of 6 months were obtained from Shanghai Jiagan Laboratory Animal Co., Ltd. and raised individually in the Animal Experiment Center of the Naval Medical University (Shanghai, China). All experimental procedures of this study were carried out in compliance with internationally accepted humane standards (Russell et al., 1959) and approved by the Ethics Committee for Animal Experiments of the university (Approval no. 20171220058). Food was given daily in the amount of 2% of their body weight, and water was available *ad libitum*. The animals were accustomed to the general experimental environment, with temperature and humidity of 23°C and 50–65%, respectively. They were singly utilized in the study and were fasted overnight before anesthetizing in the morning.

### Experimental Design

**FIGURE 1:**
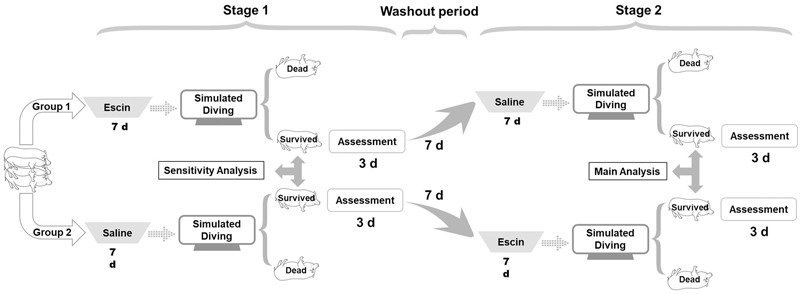
Profile of escin/saline treatment in a swine decompression sickness (DCS) model. Sixteen swine were randomly divided into two groups (8 each) and were subjected to two simulated air dives in a hyperbaric chamber with an interval of 17 days. One week before each dive, the swine were administered daily with escin or saline for 7 days. Group 1 animals received escin treatment prior to the first dive and saline prior to the second; Group 2 received saline first and then escin. Assessment of DCS was performed for 3 days following each dive.

Swine were randomly divided into two groups (*n* = 8) with the respective sequence of interventions described below. All the animals underwent surgical preparation for deep vein catheterization 3 days before the treatment. In Group 1, simulated diving was conducted after a successive daily administration of escin for 7 days, which constituted Stage 1 of the experiment. A week later, saline was administered for 7 days before a second simulated dive, which constituted Stage 2 of the experiment. The 7-day period between the two stages served as a washout period for the first-stage treatment. In Group 2, saline was administered in Stage 1 and escin in Stage 2 before the respective simulated dives (Figure 1). The administration of escin was performed by mixing 0.4 mg/kg/day of escin solution (0.16 g/L) into food, and an equal volume of saline was administrated as control. The animals were continuously observed during the experiment, and DCS was evaluated after each simulated dive.

### Surgical Preparation

The surgery procedure was the same as in a previous study (Qing et al., 2018). Briefly, after the experimental animal calmed down, anesthesia was conducted with 0.05 mg/kg atropine and 0.1 ml/kg Sumianxin intramuscularly injected with an interval of 15 min; 2 mg/kg propofol was injected intravenously, and anesthesia was maintained with inhaled 6% isoflurane using an anesthetic machine (WATO EX-20 Vet, Mindray, Shenzhen, China). A central venous catheter (REF ES-04301, Arrow, United States) was introduced into the right external jugular vein for blood sampling. After completely recovering, the animals were sent back without any further interventions that day.

### Simulated Diving

A steel animal chamber (DWC150, Yangyuan No. 701 Institute, Shanghai, China) was used for all simulated dives, one animal at a time. The chamber was pressurized to 30 msw with compressed air in 6 min at an increasing rate from 3 to 6 msw/min to minimize the possibility of middle ear squeeze. Pressure was maintained for 2 h before decompression lineally at 5 msw/min to atmospheric pressure. To prevent any decrease in O_2_ and accumulation of CO_2_, the chamber was frequently ventilated. During exposure, the temperature was maintained between 22 and 24°C with transient minor changes following compression and near surfacing (Qing et al., 2018).

### Bubble Detection

An ultrasound machine (Mylab 30CV, Esaote, Italy) with a 3.5-MHz transducer was used for detecting bubbles in heart chambers as previously described (Eftedal and Brubakk, 1997; Qing et al., 2017). Briefly, detection was repeated before each stage and at 0.5, 1, 1.5, 2, 3, 4, and 6 h after decompression, each lasting 2 min. Aortic (AO) root short axis, the best view, was adjusted for bubble detection, so that the right ventricular outflow tract (RVOT), pulmonary artery (PA), and AO could be clearly observed. Bubbles were scored by the Eftedal–Brubakk grading scale depending on the ultrasound images (Eftedal and Brubakk, 1997).

### Skin Lesion Assessment

Skin lesions were thoroughly examined after surfacing following the rules developed in our previous study (Qing et al., 2017). Briefly, the latency was recorded, and the dimensions of stage III lesions were measured by the palm of a single experimenter. Swine body surface area was calculated by the Meeh–Rubner equation *area = 0.0974 × weight^2/3^*(Quiring, 1955; Qing et al., 2018).

### Platelet Counting

Right before each stage, before hyperbaric exposure, and 6 h post decompression, blood samples were obtained from the indwelling catheter, 2 ml into an EDTA anticoagulated tube for platelet count using a hematology analyzer (BC-2800Vet, Mindray, China).

### Detection of Inflammation and Endothelial Markers

Before each stage, right before exposure, and 5 min, 1, 6, 12, 18, 24, 36, 48, and 72 h following exposure, 2 ml blood samples were taken from the catheter into pro-coagulation tubes, and serum was separated. Inflammatory indicators, including interleukin-1β (IL-1β), IL-6, and methane dicarboxylic aldehyde (MDA), and endothelial markers, including endothelin-1 (ET-1) and intercellular adhesion molecule-1 (ICAM-1), were detected by the respective enzyme-linked immunosorbent assay (ELISA) kits (Westang Biotech Inc., Shanghai, China).

### Statistical Analysis

Individuals who survived the second dive in the two groups were combined. Two-stage indexes collected from the combined group, including skin lesions, platelet count, and inflammation and endothelial markers, were compared by analysis of variance with two-stage cross-over design as the main analysis. Meanwhile, these indexes of Stage 1 between groups were compared by independent-samples *t*-test as the sensitivity analysis. Indexes of Stage 2 between the two groups were also compared by independent-samples *t*-test. The main analysis was statistically more efficient than the sensitivity analysis. Bubble loads were analyzed by the generalized estimation equation. Differences in the same treatment between groups were compared by two independent-samples *t*-tests. *P* ≤ 0.05 was accepted as statistically significant.

## Results

### General Description

The swine weighed 20.9 ± 1.3 kg (mean ± SD) at the beginning of the experiment. There was no significant difference in mean mass between the two groups before the first or the second stage. Mean body mass had increased by approximately 8% by the day of the second simulated dive compared with that of the first dive, from 21.3 ± 1.1 to 23.1 ± 1.2 kg.

A general analysis of the changes in the indicators showed that the first stage or the first dive had no effect on the second in both groups and in the combined group. No discernable circulating bubbles and skin lesions were found in any individuals who survived the first dive on the day before Stage 2. Detailed statistical results are shown in Table 1.

**Table 1:** Results of ANOVA with two-stage cross-over design and two independent-samples *t*-tests.

					Two independent-	
Parameters	Testing time	ANOVA			samples *t*-tests	
		Sources of
		variation	*F*	*P*	Groups	*P*
Skin lesion area rate	Peak	Subjects	9.986	0.003	Escin	0.687
		Stages	1.260	0.299	Saline	0.289
		Treatments	33.696	0.001	Combined	0.180
Platelet count	6 h after dive	Subjects	5.333	0.020	Escin	0.225
		Stages	0.081	0.784	Saline	0.428
		Treatments	29.517	0.001	Combined	0.125
IL-1β	Average	Subjects	2.794	0.097	Escin	0.353
		Stages	0.027	0.874	Saline	0.906
		Treatments	22.587	0.002	Combined	0.531
	Peak	Subjects	2.295	0.145	Escin	0.461
		Stages	0.012	0.917	Saline	0.811
		Treatments	30.816	0.001	Combined	0.527
IL-6	Average	Subjects	3.063	0.079	Escin	0.183
		Stages	0.721	0.424	Saline	0.964
		Treatment	8.620	0.022	Combined	0.692
	Peak	Subjects	10.212	0.003	Escin	0.377
		Stages	2.443	0.162	Saline	0.893
		Treatment	19.896	0.003	Combined	0.894
ICAM-1	Average	Subjects	13.866	0.001	Escin	0.975
		Stages	7.015	0.033	Saline	0.253
		Treatments	48.551	0.000	Combined	0.234
	Peak	Subjects	8.073	0.006	Escin	0.900
		Stages	6.913	0.034	Saline	0.193
		Treatments	45.263	0.000	Combined	0.209
ET-1	Average	Subjects	4.833	0.026	Escin	0.919
		Stages	0.032	0.862	Saline	0.688
		Treatments	9.264	0.019	Combined	0.940
	Peak	Subjects	17.266	0.001	Escin	0.596
		Stages	0.147	0.713	Saline	0.983
		Treatments	42.327	0.000	Combined	0.533
MDA	Average	Subjects	12.080	0.002	Escin	0.220
		Stages	6.610	0.037	Saline	0.925
		Treatments	12.080	0.002	Combined	0.681
	Peak	Subjects	13.554	0.001	Escin	0.686
		Stages	0.462	0.518	Saline	0.779
		Treatments	83.030	0.000	Combined	0.906

*Swine were subjected to two simulated air dives to 30 msw for 2 h following treatment with either escin or saline for 7 days. After surfacing, skin lesions were monitored, and blood was sampled for platelet count and determination of inflammatory and endothelia related indices including IL-1β (interleukin-1β), IL-6 (interleukin-6), ICAM-1 (intercellular adhesion molecule-1), ET-1 (endothelin-1), and MDA (methane dicarboxylic aldehyde). Two types of statistical analysis were used to test the effect of the first stage or the first dive on the second in both groups and after combination.*

### The Death Rate of Decompression Sickness

**FIGURE 2:**
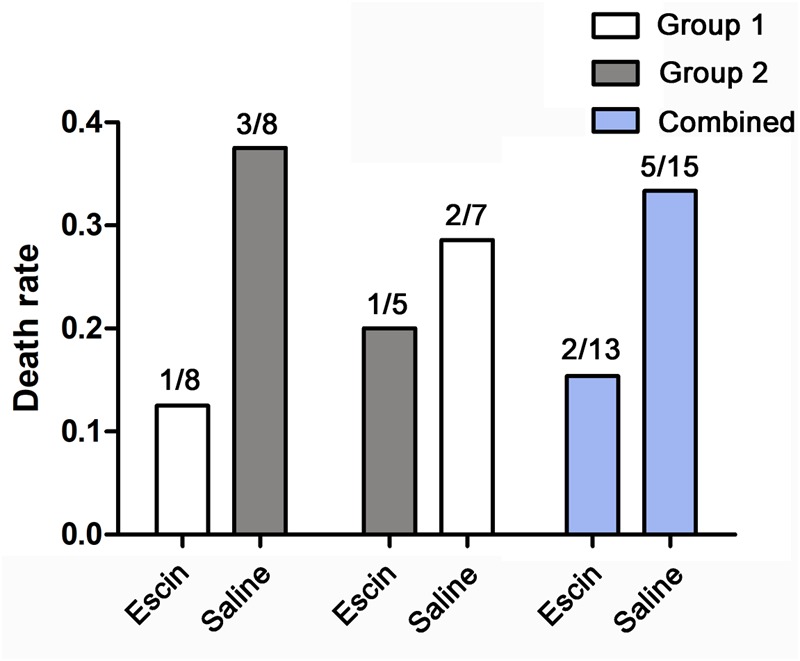
Effects of escin on the death rate of DCS in swine. Swine were subjected to two simulated air dives to 30 msw for 2 h following treatment with either escin or saline for 7 days. The death rates were lower in animals treated with escin, but statistical significance was not reached, due to the small sample size of the animals.

Death is a common and unpredictable outcome in DCS experimental studies (Spiess et al., 2009; Mahon et al., 2014). The death rates were 1/8 and 2/7 in Group 1 and 3/8 and 1/5 in Group 2 for the first and second dive, respectively. Although statistical significance is limited by the number of animals, the death rate was lower in the escin groups than in saline-treated swine (2/13 vs. 5/15) (Figure 2). Death generally occurred 20–30 min after surfacing with sudden stiffness and convulsion after severe breathing difficulty, which lasted for around 1 min and was too short to take steps to alleviate any possible discomfort.

### Bubble Load Following Escin Treatment

**FIGURE 3:**
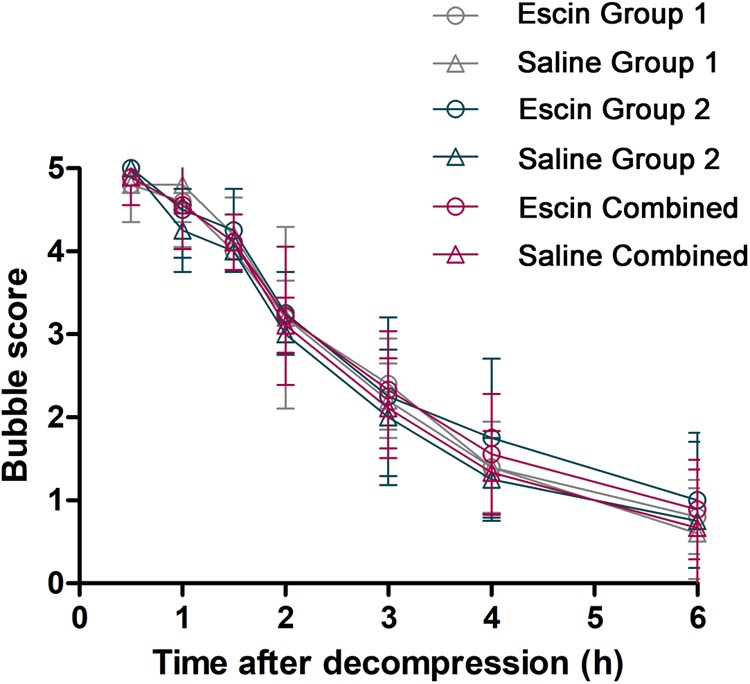
Bubble load in swine after simulated diving following escin treatment. Swine were divided into two groups (*n* = 8) and were subjected to two simulated dives following treatment with either escin or saline (see Figure 1). Bubbles were detected by ultrasound at the time points after decompression and were scored by the EB grading scale. No statistical variance was found between the groups.

Bubbles could be clearly observed in the RVOT and PA on ultrasonoscopy. The number of bubbles peaked when the first detection was performed 30 min after surfacing, and gradually decreased thereafter (Figure 3). Escin had no effect on bubble load compared with saline.

### Effects of Escin on Skin Lesions

**FIGURE 4:**
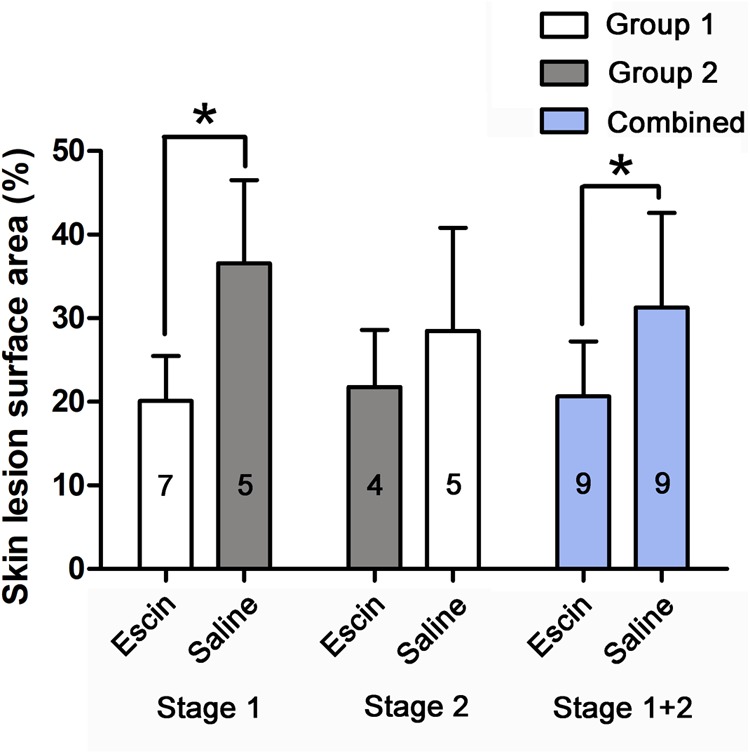
Skin DCS lesions in swine after simulated diving following escin treatment. Skin lesions were observed in swine after simulated air dives following escin or saline treatment. The skin lesion as a proportion of body surface area was compared between different treatments. The values in the chart bar represent the number of animals. ^∗^*P* < 0.01.

Skin lesions were observed in all swine after either of the simulated air dives. Escin decreased the lesion rate by 16.4 and 10.6% in Stage 1 and in the combined group, respectively (*P* < 0.01), and by 8.3% in Stage 2 (*P* = 0.365), when compared between the two groups (Figure 4).

### Effects of Escin on Platelet Count in Decompression Sickness Swine

**FIGURE 5:**
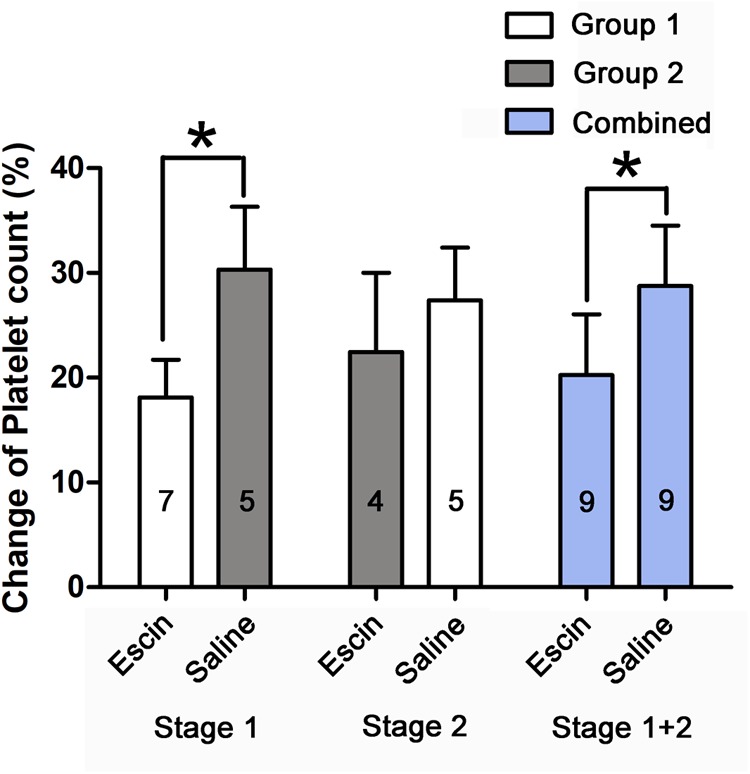
Effects of escin on platelet count in DCS swine. Blood was sampled from a venous catheter in swine treated with saline or escin before simulated air diving and 6 h following decompression. The rate of change in platelet count was compared between different treatments. The values in the chart bar represent the number of animals. ^∗^*P* < 0.01.

The simulated dives induced a significant decrease in platelet count. Escin attenuated the level of decrease by about 8.5 and 11.2% in Stage 1 and the combined group, respectively, and 4.9% in Stage 2 (*P* = 0.277), as shown in Figure 5.

### Anti-inflammatory Effects of Escin on Decompression Sickness Swine

**FIGURE 6:**
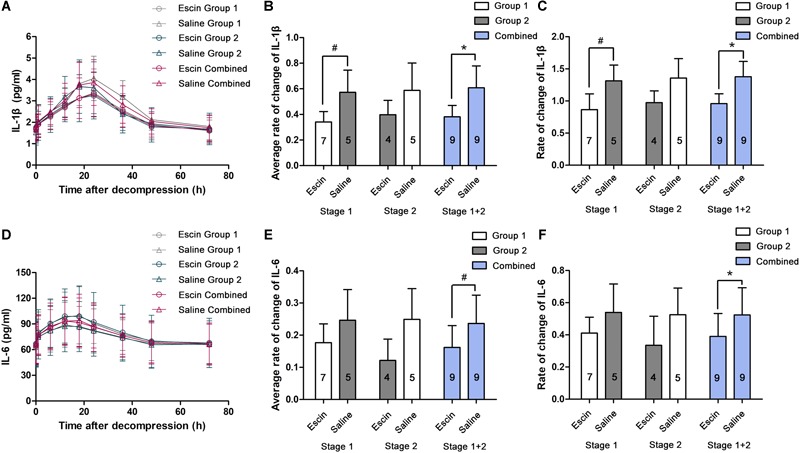
Effects of escin on inflammatory indicators in DCS swine. Blood was sampled from a venous catheter in swine treated with escin or saline before a simulated air dive and 5 min, 1, 6, 12, 18, 24, 36, 48, and 72 h following decompression. Interleukin-1β (IL-1β; **(A)** and interleukin-6 (IL-6; **D**) in serum were tested by enzyme-linked immunosorbent assay (ELISA). The rates of changes were compared between treatments in average levels **(B,E)** and at the peak time points **(C,F)**. The values in the chart bar represent the number of animals. ^∗^*P* < 0.01, ^#^*P* < 0.05.

IL-1β and IL-6 gradually increased and peaked at 24 and 12 h after the simulated dives, respectively (Figure 6A,D). The rates of change were compared between the different treatments in average levels and at their peak time points. Escin significantly reduced the increase in IL-1β level in Stage 1 and the combined group and IL-6 level in the combined group in average levels and at their peak time points. There was no statistical difference in IL-1β in Stage 2 and IL-6 in Stages 1 and 2, as shown in Figure 6B,C,E,F.

### Effects of Escin on Endothelial and Anti-oxidative Indices in Decompression Sickness

**FIGURE 7:**
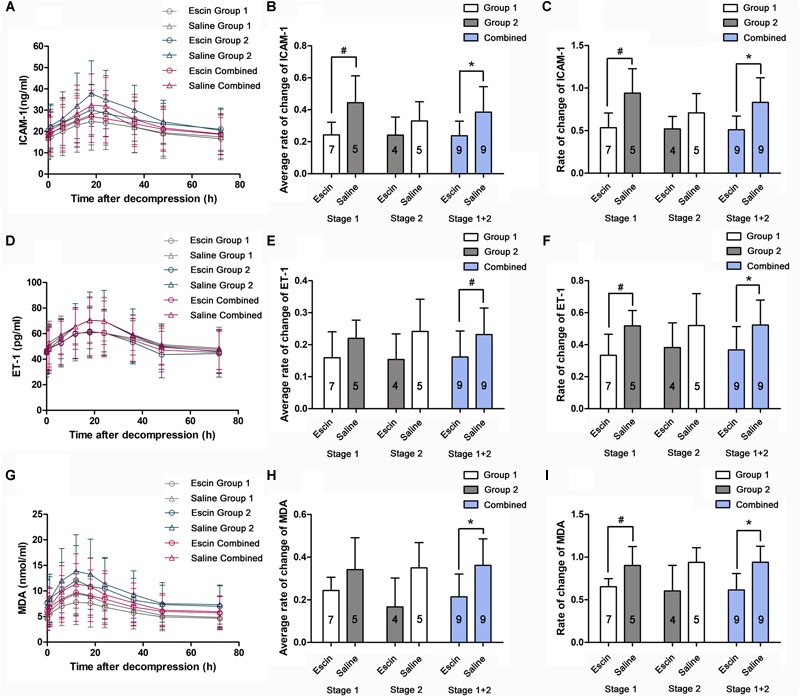
Effects of escin on endothelial and anti-oxidative indices in DCS swine. Blood samples were obtained from a venous catheter in swine treated with escin or saline before a simulated air dive and at 5 min, 1, 6, 12, 18, 24, 36, 48, and 72 h following decompression. Serum intercellular adhesion molecule-1 (ICAM-1; **A**), endothelin-1 (ET-1; **D**), and methane dicarboxylic aldehyde (MDA, **G**) levels were tested by ELISA. The rates of changes were compared between treatments in average levels **(B,E,H)** and at the peak time points **(C,F,I)**. The values in the chart bar represent the number of animals. ^∗^*P* < 0.01, ^#^*P* < 0.05.

A significant increase of serum ICAM-1, ET-1, and MDA was induced after the simulated dives, with the peak value appearing at 18, 18, and 12 h, respectively (Figure 7A,D,G). The rates of change were compared between the different treatments in average levels and at their peak time points. Escin reduced the increase of ET-1 and MDA level in the combined group (Figure 7E,H) and ICAM-1 level in Stage 1 and the combined group (Figure 7B) in average levels. Escin significantly reduced the levels of these markers at the respective peak time point in both Stage 1 and the combined group (Figure 7C,F,I). There was no statistically significant difference in these markers following Stage 2 in average levels and at their peak time points, and no statistically significant difference in ET-1 and MDA in Stage 1 in average levels (Figure 7E,H).

## Discussion

Improper rapid decompression after hyperbaric exposure increases the risk of DCS, a distinctive disorder associated with bubble generation in the body. The symptoms vary from mild arthralgia and skin lesions to severe cardio-pulmonary dysfunction or neurological impairment, and even sudden death (Vann et al., 2011). Invisible injuries, including inflammatory responses and vascular endothelial injuries, also play a role in the progress of DCS (Bigley et al., 2008; Steyers and Miller, 2014). Bubbles in vessels induce mechanical damage when in direct contact with endothelium. Oxidative stress and other diving-induced insults by circulating bubbles are also involved in the pathogenesis of endothelial dysfunction (Thom et al., 2011; Wang et al., 2015; Zhang et al., 2017b). Approaches to protect endothelium represent a promising potential possibility to prevent or alleviate DCS.

Based on our previous confirmation of the protective effects of escin using a DCS rat model (Zhang et al., 2017a), a swine model was adopted in this study to further verify the beneficial effects. The genetic structure and body size of swine are closer to those of humans when compared with rats. Highly visible DCS signs such as pronounced cutaneous lesions together with the advantages in repeated sampling make this study design better suited to DCS research (Qing et al., 2017). In addition, before trialing escin in human divers, it is necessary to undergo verification in a larger animal model (Swindle et al., 2012).

In this study, a two-stage experimental design was adopted, which increased the validity through self-comparison of the two different treatments. Meanwhile, the number of animals used was minimized through increased statistical efficiency. An effective washout period is a prerequisite for applying the two-stage cross-over design to DCS research, which avoids any influence of the first treatment upon the second. An interval of 7 days was employed, and comparing the various indicators between the beginnings of each stage indicated the sufficiency of this washout period, which was proven in a previous swine DCS study (Qing et al., 2018). The data loss caused by the death of individuals was minimized, and the survivors of the second stage were used for the main analysis. The survivors of the first stage were used for sensitivity analysis to further verify the reliability of the main analysis. To ensure that the washout period was adequate, sensitivity analysis can be used as a separate confirmation of the results (Thabane et al., 2013). The second phase used the survivors from the first phase, and thus, randomization was compromised; therefore, inter-group comparisons in the second stage would not have been statistically reasonable. The presentation of Stage 2 results in the figures is merely an objective representation of the results.

As mentioned in our previous study, the temporal characteristics of DCS together with the metabolic half-life of escin (8–12 h) make escin suitable for both pre-dive and post-DCS administration (Zhang et al., 2017a). Meanwhile, the therapeutic effects remain to be explored. In the present study, the employment of a successive 7-day pre-dive dosing regimen was intended to better demonstrate the beneficial effects of escin. The dosage in this study was calculated in accordance with the equivalent dose conversion from different species to human beings (Tiffany et al., 2002).

Escin might protect the vascular endothelia directly or indirectly through several pharmacological mechanisms and, hence, may effectively relieve multiple injuries of DCS (Sirtori, 2001; Zhang et al., 2017a). Bubbles in circulation can act on the endothelial lining of vessels directly or *via* increased shear stress, bring about perturbations, induce activation, or even strip away endothelial cells (Brubakk and Møllerløkken, 2009; Zhang et al., 2016). Escin could alter the skeletal structure of endothelial cells, enhance intercellular connectivity, and inhibit the degradation of proteoglycans in endothelial cells (Vašková et al., 2015; Domanski et al., 2016). Escin could also relieve the injury of blood vessel walls by stabilizing the lysosomal membrane, inhibiting serum lysosomal activity and protease metabolism (Sirtori, 2001; Domanski et al., 2016). Both pathways ultimately reduce capillary permeability and inflammatory exudation encountered in DCS. The results in the present study are in accordance with these mechanisms.

Platelet activation by bubbles and damaged endothelial cells is a probable key contributor in the pathogenesis of DCS (James et al., 2003). Escin was demonstrated in this study to decrease platelet aggregation in DCS. Because no change was shown in the circulating bubbles, the effects on platelets were more likely related to endothelial protection. Another effect of escin is to alleviate venous stasis by reducing thrombosis and acting on vascular endothelial cell receptors, causing venous contraction, increasing the elasticity and tension of the venous wall, which results in a speed-up of venous return and reduction of venous volume and pressure (Domanski et al., 2016). Bubble-induced venous stasis and inflammatory exudation are the primary pathogenesis of skin lesion in DCS (Qing et al., 2017). The significant alleviation of skin lesion surface area in the present experiment supports the mechanisms described above.

Meanwhile, levels of IL-1β and IL-6 and activity of MDA significantly decreased in escin-treated DCS swine in this study. The beneficial effects showed another anti-inflammatory property of escin, which may still rest partly on its endothelial protection. Escin has been found to inhibit neutrophil infiltration, lipid peroxidation, and inflammatory mediator release, and to improve the activity of superoxide dismutase (SOD) and glutathione (Sirtori, 2001).

The inflammatory and endothelial indicators in this study were mostly selected based on the results from our previous study in a DCS rat model (Zhang et al., 2017a). With the exception of TNF-α and E-selectin, which showed no change in our DCS swine model, all the other markers in the current study presented similar changes to those from rats. However, the peak and recovery times were different; the underlying reasons for these differences deserve further study. Nevertheless, the endothelial indicators were proven again to act as biomarkers of DCS, and the time courses of the evolution could serve as a diagnostic index to evaluate the severity of DCS at different time points following pathogenesis (Zhang et al., 2017b). Though ICAM-1 and ET-1 are widely considered as markers of endothelial cell activation, they indeed are originated from many cells including endothelial cells. In DCS, the pathogenesis of circulating bubbles and the results of endothelial protection suggest that the changes in some indicators may be more closely related to the endothelium, but would never rule out other sources. Because of the sample size, statistical analysis of the death rate was non-significant, despite the positive trend showing a benefit from escin treatment. There was a 17-day interval between the two dives, and animal weight increased by an average of 8%. Body weight may affect DCS, but the crossover design diminished the potential influence of this possible confounder. Simultaneously, comparison between the two stages in both groups and after combination of the two groups shows no obvious difference, with the same death rate of 25%. In addition, accelerated venous flow reflux might accelerate the removal of supersaturated inert gasses during and after decompression, and increased activity of endothelial nitric oxide synthase (eNOS) by escin might reduce the bubble formation by facilitating the elimination of vascular gaseous nuclei (Wisløff et al., 2004). On the other hand, the vasoconstriction property of escin may delay the elimination of inert gasses. Although no effect on bubble formation was observed in this study, the possible interaction with inert gas removal deserves further exploration in future studies. Furthermore, the mechanism of escin against DCS on the molecular and cellular levels also needs studies.

Taken together, this is the first study revealing that escin significantly reduced the risk of DCS in a swine model. Endothelial-protective and anti-inflammatory properties might be the underlying mechanism. Though there still is a lack of high-quality clinical trials to confirm its effectiveness, escin is pharmacologically mature with few side effects and may be served as a candidate drug in DCS prevention or treatment after additional experimental support.

## Data Availability

The raw data supporting the conclusions of this manuscript will be made available by the authors, without undue reservation, to any qualified researcher.

## Ethics Statement

All experimental procedures of this study were carried out in compliance with internationally accepted humane standards and approved by the Ethics Committee for Animal Experiments of the Naval Medical University.

## Author Contributions

W-gX, LQ, WM, and WZ designed the experiments. LQ, WM, WZ, and H-jY conducted the experiments. LQ, WM, WZ, H-jY, KZ, DA, and W-gX contributed to data analyses and interpretation of the results. W-gX, LQ, WM, and WZ wrote the manuscript and prepared all the figures and the table. KZ and DA revised the manuscript.

## Conflict of Interest Statement

The authors declare that the research was conducted in the absence of any commercial or financial relationships that could be construed as a potential conflict of interest.
