# Corrigendum: Benefits of Escin for Decompression Sickness in Bama Pigs by Endothelial-Targeting Protection

**DOI:** 10.3389/fphys.2019.00748

**Published:** 2019-06-20

**Authors:** Long Qing, Wentao Meng, Wei Zhang, Hong-jie Yi, Kun Zhang, Dinesh K. Ariyadewa, Wei-gang Xu

**Affiliations:** ^1^Department of Diving and Hyperbaric Medicine, Naval Medical University, Shanghai, China; ^2^Department of Medicine, Sri Lankan Navy, Colombo, Sri Lanka

**Keywords:** decompression sickness, horse chestnut seed, escin, swine, endothelial dysfunction

In the original article, there were mistakes in the legends for **Figure 2** and **Table 1** as published. The value of the hyperbaric exposure time (2 h) is incorrectly written as the value of the decompression time (6 h). The correct legend appears below. The authors apologize for this error and state that this does not change the scientific conclusions of the article in any way. The original article has been updated.

**Figure 2** | Effects of escin on the death rate of DCS in swine. Swine were subjected to two simulated air dives to 30 msw for 2 h following treatment with either escin or saline for 7 days. The death rates were lower in animals treated with escin, but statistical significance was not reached, due to the small sample size of the animals.

**Table 1**| Swine were subjected to two simulated air dives to 30 msw for 2 h following treatment with either escin or saline for 7 days. After surfacing, skin lesions were monitored, and blood was sampled for platelet count and determination of inflammatory and endothelia related indices including IL-1β (interleukin-1β), IL-6 (interleukin-6), ICAM-1 (intercellular adhesion molecule-1), ET-1 (endothelin-1), and MDA (methane dicarboxylic aldehyde). Two types of statistical analysis were used to test the effect of the first stage or the first dive on the second in both groups and after combination.

